# Reusable, Non-Invasive, and Ultrafast Radio Frequency Biosensor Based on Optimized Integrated Passive Device Fabrication Process for Quantitative Detection of Glucose Levels

**DOI:** 10.3390/s20061565

**Published:** 2020-03-11

**Authors:** Yang Li, Zhao Yao, Wenjing Yue, Chunwei Zhang, Song Gao, Cong Wang

**Affiliations:** 1 School of Information Science and Engineering, University of Jinan, Jinan 250022, China; ise_liy@ujn.edu.cn (Y.L.); ise_zhangcw@ujn.edu.cn (C.Z.); 2 Shandong Provincial Key Laboratory of Network Based Intelligent Computing, University of Jinan, Jinan 250022, China; 3 College of Microtechnology & Nanotechnology, Qingdao University, Qingdao 266071, China; yao9074@hotmail.com; 4 Department of Microwave Engineering, Harbin Institute of Technology, Harbin 150001, China; kevinwang@hit.edu.cn

**Keywords:** radio frequency resonator, non-invasive glucose biosensor, integrated passive device fabrication process, quantitative detection, reusable biosensor, ultrafast biosensor

## Abstract

The increase in the number of people suffering diabetes has been the driving force behind the development of glucose sensors to overcome the current testing shortcomings. In this work, a reusable, non-invasive and ultrafast radio frequency biosensor based on optimized integrated passive device fabrication process for quantitative detection of glucose level was developed. With the aid of the novel biosensor design with hammer-shaped capacitors for carrying out detection, both the resonance frequency and magnitude of reflection coefficient can be applied to map the different glucose levels. Meanwhile, the corresponding fabrication process was developed, providing an approach for achieving quantitative detection and a structure without metal-insulator-metal type capacitor that realizes low cost and high reliability. To enhance the sensitivity of biosensor, a 3-min dry etching treatment based on chlorine/argon-based plasma was implemented for realizing hydrophilicity of capacitor surface to ensure that the biosensor can be touched rapidly with glucose. Based on above implementation, a non-invasive biosensor having an ultrafast response time of superior to 0.85 s, ultralow LOD of 8.01 mg/dL and excellent reusability verified through five sets of measurements are realized. The proposed approaches are not limited the development of a stable and accurate platform for the detection of glucose levels but also presents a scheme toward the detection of glucose levels in human serum.

## 1. Introduction

Diabetes is a group of lifelong metabolic diseases caused by multiple etiologies, which is characterized by chronic hyperglycemia. Human in hyperglycemic for long-term will lead to microvascular damage and endanger the heart, brain, kidney, peripheral nerves, eyes, feet, etc. [1]. According to the World Health Organization statistics, diabetes, having more than 100 diabetes complications is one of the most complicated diseases. It is known that more than half of diabetic deaths are caused by cardiovascular and cerebrovascular diseases, and 10% are caused by nephropathy [2,3,4,5]. Patients with amputation due to diabetes are 10 to 20 times more numerous than non-diabetic patients. Clinical data show that 30~40% of patients have at least one complication after the onset of diabetes around 10 years, and drug treatment is difficult to recover since complications occur [6]. Hence, it is crucial to develop a method to realize accurate glucose detection for treatment and management of diabetes. The glucose biosensing detection system composed of bio-sensing detection conversion and peripheral electronic systems can be applied to monitor the glucose concentration of diabetic patients in time. In particular, biosensing detection conversion plays a very important role in the sensing system, which determines several key factors, such as accuracy, detection time and system cost [7,8,9]. A biosensor can convert different concentrations of glucose into corresponding electrical signals for output, which has advantages of high accuracy, fast analysis, low cost, good repeatability, simple operation and high specificity by comparing with traditional detection methods [10,11,12,13,14,15,16]. With the aid of chips, microfluidics systems and labs-on-a-chip, biosensor technology have developed rapidly and can be divided into electrochemical biosensors and optical biosensors based on the type of signal conversion [17,18,19,20,21,22,23,24,25,26]. Electrochemical biosensors enable the detection of biomarkers by adding specific enzymes on the electrodes, which has many advantages, such as high sensitivity, simple operation and cost-effective [27,28,29]. However, the introduction of some small molecule exotic vectors acting as channels between enzymes and motors electrodes leads to a slowing reaction, a decreasing performance, a deteriorating reliability and frequent replacement. Moreover, another key factor limiting the application of electrochemical sensors is the fact that the electrolytes need to be replenished on a regular basis, which significantly increase burden to subsequent costs [30,31,32]. With regard to optical biosensors, it needs a long settling time for detection and is highly susceptible to change the test results for ambient light [33,34,35,36,37,38,39,40,41]. Recently, a biosensor based on radio frequency (RF) techniques has attracted enormous attention and is regarded as a promising and competitive candidate for implementing third-generation glucose biosensors [42,43,44,45,46,47]. Compared to other types of biosensors, RF biosensors offer the following advantages: first, they are different from the electrochemical biosensors, which are subject to the constraints of the use environment and performance degradation with service time. The RF biosensors are not susceptible to environmental factors such as external light, temperature, humidity, etc., and can maintain stability in long-term complex environments. Secondly, The RF biosensor can perform rapid detection of biomarkers in real time without the need for pre-stabilization time. Thirdly, the detection time of the RF biosensor only depends on the sweep period of the vector network analyzer, exhibiting an evident advantage in short detection time. Fourthly, there is no need to add exotic vectors to mark for sensing and a label-free detection is achieved by dropping the determinand to the RF biosensor detection area.

In this work, we developed a non-invasive RF glucose sensor based on quantitative detection. Benefiting from the novel biosensor design with hammer-shaped capacitors, it was found that the concentrations of glucose are proportional to the biosensor resonance frequency and magnitude of reflection coefficient, which implies that both the resonance frequency and the magnitude of reflection coefficient can be applied to map the different glucose levels, indicating the feasibility of the proposed biosensor in glucose detection. Moreover, the corresponding fabrication process has been further elaborated, holding several merits such as that it can provide a volume-fixed passivation layer for quantitative detection and non-MIM (metal-insulator-metal)-type capacitor for reducing fabrication procedure with low cost and high reliability. To enhance the sensitivity of a biosensor, a dry etching treatment was implemented by using chlorine/argon-based plasma for realizing hydrophilicity of capacitor surface to ensure that the biosensor can be touched rapidly with glucose. Ultimately, a non-invasive biosensor with a memorable response time, ultralow limit of detection (LOD) and excellent reusability were realized and verified through measurements.

## 2. Materials and Methods

### 2.1. Biosensor Operating Mechanism

**Figure 1:**
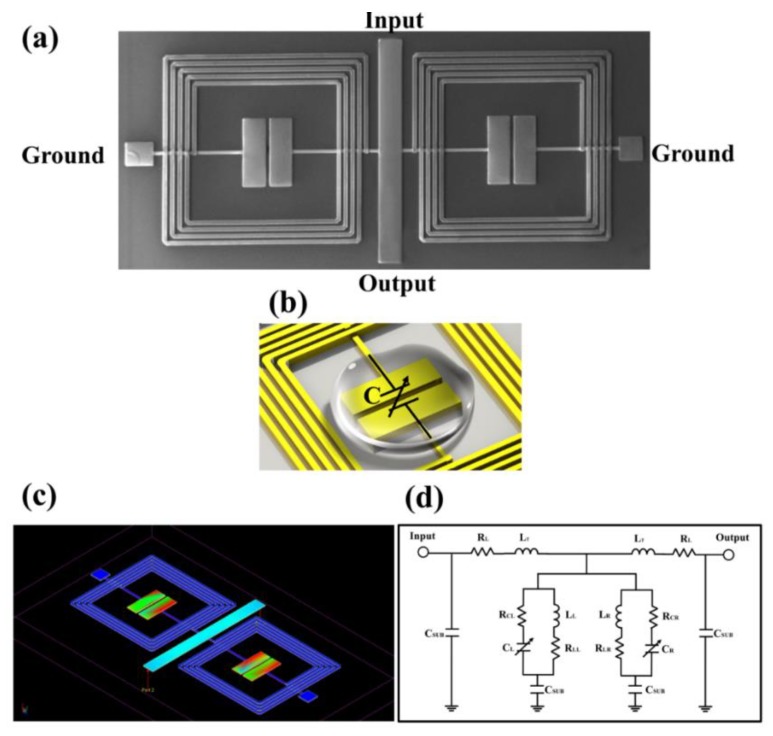
The illustration of glucose biosensor based on the RF resonator. (**a**) The SEM image of the fabricated biosensor with ports description. (**b**) The sensitive area of the hammer-shaped capacitor. (**c**) The simulated electric field distribution for the proposed biosensor at the resonance frequency. (**d**) The equivalent circuit model of the proposed biosensor.

The scanning electron microscope (SEM) image of the proposed glucose biosensor based on RF resonator is illustrated in Figure 1a, which is composed of two spiral inductors and two hammer-shaped capacitors. An embedded structure was skillfully introduced to minimize the dimensions of the overall circuit. To form inductor-capacitor (L-C) resonance tanks, two capacitors were placed in parallel on two inductors and two common ports for connecting inductor and capacitor were defined as ground. For the detailed parameters of the proposed pattern, the two inductors metal lines were 15 µm in width, the turns were 4.5, the inner diameter was 610 µm, and the spacing of the metal line is 15 µm. As regards the hammer-shaped capacitors, it consisted of two rectangles with a length of 300 µm and a width of 95 µm. In order to form a capacitor, the distance between the two rectangles was set to 20 µm. After dicing, the total area of the chip was 1060 µm × 2380 µm. The microwave propagation theory can be applied to analyze the biosensor operating mechanism, described as follows. The RF signal is initiated at the input port, transmitted along the middle straight transmission line, partially attenuated at both L-C-type resonators, ending at the output port. Specifically, the two hammer-shaped capacitors are the key parts of detection, which are employed to alter the capacitive effect. As shown in Figure 1b, when the glucose samples are added onto the circuit, the performance of the circuit will be changed due to the alterative capacitance, that is, because different glucose concentrations have a different permittivity to affect the capacitance. Therefore, glucose concentrations at different levels can be detected by analyzing relevant variation of RF parameters, such as magnitude of reflection coefficient and frequency shift, indicating that such a chip can be used clinically to diagnose blood glucose concentrations in diabetic patients in real time. Then, an electric field distribution of the proposed biosensor is simulated based on an Advanced Design System Momentum Microwave simulator at the resonance frequency, as shown in Figure 1c. It can be observed that the maxima of the electric field is located along two inner capacitors, which means that the electric coupling of the two capacitors plays the dominant role in the resonance frequency, validating the correctness of the above analysis. Figure 1d shows the equivalent circuit modelling of the circuit. It can be observed that the equivalent circuit consists of two L-C resonance tanks, which, respectively, correspond to the resonance tank composed of a spiral inductor and a hammer-shaped capacitor in parallel. With regard to the detailed symbols, they are depicted as follows: L_T_, R_T_, C_L_, R_CL_, L_L_, R_LL_, C_R_, R_CR_, L_R_, R_LR_ and C_SUB_ represent the inductance of the middle transmission line of the biosensor, the resistive loss of the middle transmission line of the biosensor, the capacitance of the left-side hammer-shaped capacitor, the resistive loss of the left-side hammer-shaped capacitor, the inductance of the left-side spiral inductor, the resistive loss of the left-side spiral inductor, the capacitance of the right-side hammer-shaped capacitor, the resistive loss of the right-side hammer-shaped capacitor, the inductance of the right-side spiral inductor, the resistive loss of the right-side spiral inductor, and the capacitances associated with the substrate, respectively. The resonance frequency of the proposed biosensor can be described by [48]

(1) $$f=\frac{1}{2\pi \sqrt{LC}}$$

where *C* is equal to *C_L_* and *C_R_*, which is determined by

(2) $$C=\frac{1}{2}w{\left(\frac{\varepsilon_{d}\varepsilon_{0}\varepsilon_{r}}{9.6}\right)}^{0.8}{\left(\frac{s}{w}\right)}^{m_{0}}exp\left(k_{0}\right)-3w{\left(\frac{\varepsilon_{d}\varepsilon_{0}\varepsilon_{r}}{9.6}\right)}^{0.9}{\left(\frac{s}{w}\right)}^{m_{e}}exp\left(k_{e}\right)$$

With

(3) $$m_{0}=\frac{w}{h}\left[0.619\log\left(w/h\right)-0.3853\right]$$

(4) $$k_{0}=4.26-1.453log\left(w/h\right)$$

(5) $$m_{e}=0.8675$$

(6) $$k_{e}=2.043{\left(\frac{w}{h}\right)}^{0.12}$$

where *w* is the hammer-shaped capacitor width, *h* is the thickness of GaAs substrate, *s* is the space between two capacitors, $\varepsilon_{0}$ is the free-space permittivity, $\varepsilon_{r}$ is permittivity of GaAs substrate, and $\varepsilon_{d}$ is the permittivity of the determinant. Based on Equations (2)–(6), the resonance frequency of proposed biosensor can be reproduced as

(7) $$f=\frac{1}{2\pi }L^{-\frac{1}{2}}(1/2w{\left(\frac{\varepsilon_{d}\varepsilon_{0}\varepsilon_{r}}{9.6}\right)}^{0.8}{\left(\frac{s}{w}\right)}^{m_{0}}exp\left(k_{0}\right)-3w{\left(\frac{\varepsilon_{d}\varepsilon_{0}\varepsilon_{r}}{9.6}{)}^{0.9}{\left(\frac{s}{w}\right)}^{m_{e}}exp\left(k_{e}\right)\right)}^{-\frac{1}{2}}$$

Since the design topology is fixed, the value of *L*, *w*, s, $m_{0}$, $k_{0}$, $m_{e}$, $k_{e}$, $\varepsilon_{r}$ is determined accordingly. Therefore, it can be inferred that the resonance frequency of the proposed biosensor is proportional to the permittivity of glucose solution $\varepsilon_{d}$. With regard to the reflection coefficient, it is mainly depended on the match between input impedance and characteristic impedance. In this work, the input impedance is fixed at 50 Ω, and a low reflection coefficient will be obtained if characteristic impedance *Z_c_* gets closer to 50 Ω. Here,

(8) $$Z_{c}=\frac{\eta }{2\pi \sqrt{\varepsilon_{r}}}In\left(\frac{8h}{w}+0.25\frac{w}{h}\right)$$

(9) $$\eta =\frac{12\pi }{5}\varepsilon_{d}\cdot Z_{0}$$

where *η* is the wave impedance in the glucose solution and $Z_{0}$ is the source/load impedance, the value of which is 50 Ω. Based on Equations (8) and (9), the characteristic impedance *Z_c_* can be reproduced as

(10) $$Z_{c}=\frac{6\varepsilon_{d}\cdot Z_{0}}{5\sqrt{\varepsilon_{r}}}In\left(\frac{8h}{w}+0.25\frac{w}{h}\right)$$

Based on Equation (10), we can conclude that the different glucose concentrations can also be reflected by the variation of reflection coefficient.

### 2.2. Biosensor Fabrication Techniques

This section provides a detailed explanation to the proposed biosensor fabrication processing, which is an optimized process of the traditional integrated passive device process [49,50], exploiting the hammer-shaped capacitors instead of metal-insulator-metal (MIM) capacitors. The proposed implementation not only avoids the multi-processes realization of silicon nitride (SiN_x_) based on plasma enhanced chemical vapor deposition (PECVD) method for a cost-effective fabrication, it also effectively averts the introduction of the low breakdown voltage of MIM capacitors, which is an upgrade on the reliability of the biosensor. In this work, a GaAs wafer with a relative permittivity of 12.85 and loss tangent of 0.002 was selected to fabricate biosensor, which can avoid parasitic capacitance and inductive loading of conductive substrates. The proposed fabrication process starts from substrate cleaning with an ultrasonic acetone bath (5 min), implementing isopropyl alcohol (IPA) treatment (1 min), and DI water flushing (5 min), respectively (Step 1). A passivation layer was then deposited by PECVD, comprising SiN_x_ with a relative dielectric constant of 7.5 and a loss tangent of 0.002 (step 2). The deposition was carried out at a ratio of SiH_4_ and NH_3_ of 320:9 sccm at a chamber pressure of 1200 mTorr, a chamber temperature of 250 °C, a 100 W RF power and a 2000 sccm gas flow. The entire process time was fixed to 400 s to get a target thickness of 0.2 μm. The deposited SiN_x_ layer is necessary to obtain a uniform surface on the defects and roughness of the substrate. The deposited SiN_x_ passivation layer also promotes adhesion between the bottom metal layer and the GaAs substrate. A photolithography process of the bottom metal, which involves photoresist spin coating with edge bead removal, exposure with the aid of mask, development, inspection, and critical dimension (CD) measure, was performed after SiN_x_ deposition (Step 3). According to the specific bottom metal shape desired, this work chose to perform photolithography with a negative photoresist (NR9-3000PY, Futurrex, Franklin, NJ, USA). Then a 2-µm-thick Au bottom metal layer was formed using by the sputtering deposition method (Step 4). The deposition process was performed at a pressure of 7.5 mTorr and with a minimum deposition rate of 0.5 A/s. In addition, the room temperature sputtering method was performed to achieve a better metal roughness with a RMS value of 1.93 nm, the purpose of which is to reduce the possibility of a short circuit between the bottom metal and the top metal. Then a photoresist liff-off process was carried out by spraying acetone (Step 5).

**Figure 2:**
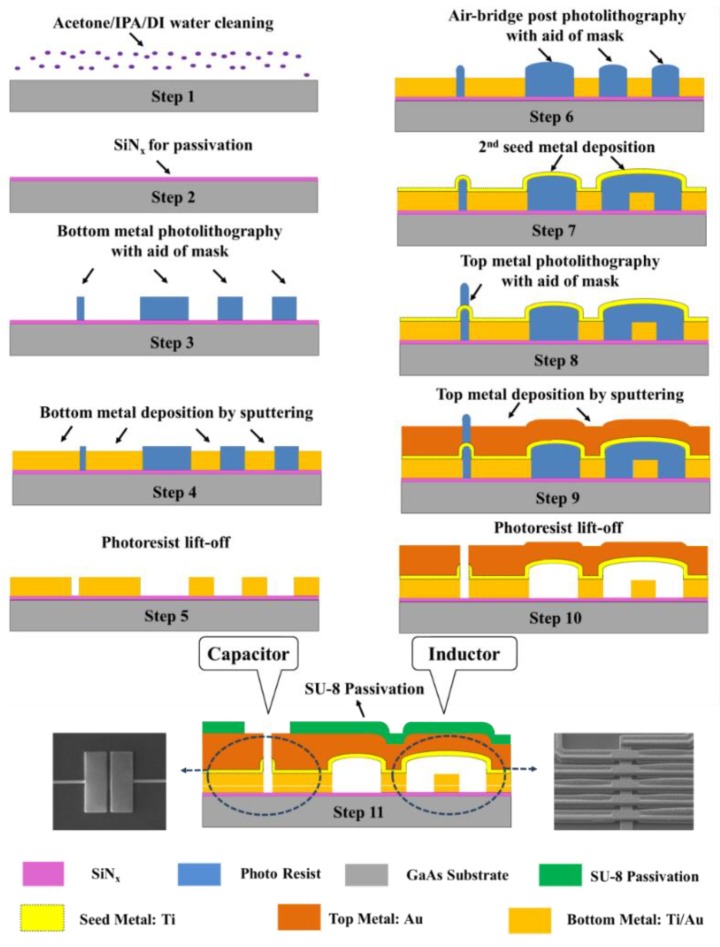
The fabrication process flow of the proposed biosensor.

Next, the forming process on the air-bridge post of spiral inductor was implemented (Step 6). First, the photolithography process, including photoresist spin coating, exposure with the aid of mask, development, inspection, CD measure, was performed using the negative photoresist, which is the same as Step 3. Then, for the purpose of constructing an arch-bridge structure, a hard-baking process followed by a photolithography process was performed, whose baking time and baking temperature were chosen as 180 s and 130 °C, respectively. Next, a Ti seed metal layer was grown by the sputtering deposition method to a thickness of 1000 nm (Step 7). An additional 30 s sputter-etching process was performed prior to Ti seed metal deposition so as to reduce the probability of occurrence of peeling off between top layer and bottom layer. Then, a top metal layer photolithography process (Step 8) and a top metal deposition (Step 9) were carried out, which are the same as step 3 and step 4. A 2 μm top metal layer was deposited using the sputtering method based on Au material, which can prevent moisture and oxidation of glucose due to its chemical stability. Subsequently, a photoresist liff-off process was carried out by spraying acetone, as in process step 5 (Step 10). Finally, a 2 µm SU-8 final passivation layer was patterned for quantitative detection to reduce the interference of the glucose solution toward the inductor and transmission-line area (Step 11). Figure 2 illustrates the whole fabrication process flow as explained above, and the inset shows SEM images of the fabricated inductor and capacitor. It should be noted that Au w chosen as the structural layer in this fabrication process, the purpose of which is as follows: first, it can prevent the metal layer from chemically reacting with substances such as glucose and water which introduce interference in repeat measurement. In addition, it is different from the conventional copper used as the metal layer; the utilization of Au layer can reduce unnecessary signal losses, which improves the transmission parameter by minimizing the signal loss.

### 2.3. RF Detection Methods

**Figure 3:**
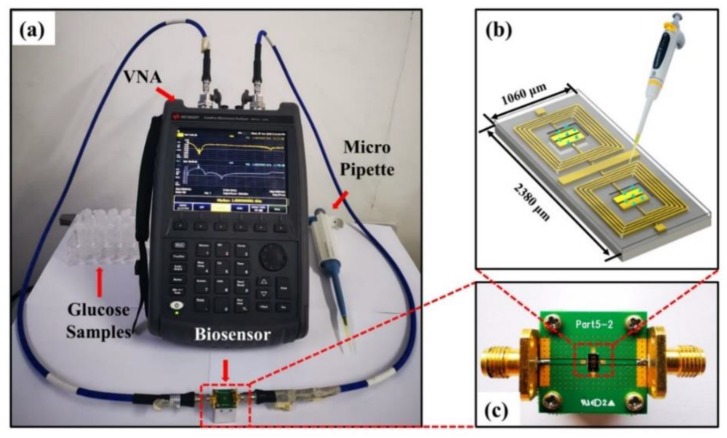
(**a**) The experimental setup of the proposed chip measurement for detecting variable glucose level. (**b**) The schematic diagram of measurement. (**c**) The fabricated biosensor attached on a PCB with input and output connection for glucose level measurement.

In order to perform the RF response measurements on the proposed biosensor, the measurement platform was successfully constructed and consisted of a vector network analyzer (VNA), a biosensor under test, a quantitative micro pipette, and glucose samples, as shown in Figure 3a. In this work, ten different glucose samples with a concentration range of 50 to 500 mg/dL were prepared, which were composed of a mixture of glucose anhydrose (Shanghai Macklin Biochemical Co., Ltd., Shanghai, China) and deionized water, and all analyzed *S*-parameters were measured using a Keysight FieldFox N9917A VNA. The proposed biosensor was attached on a printed circuit board (PCB) with two 50 Ω transmission-line ports connecting from the input to output side and two bonding-wire connecting to ground signal, as depicted in Figure 3c. The relevant RF parameters of the bare chip were collected prior to testing the target substance; afterwards, the glucose solution of different concentrations was dropped onto the test area of the biosensor through a pipette with a fixed capacity of 0.5 µL, as illustrated in Figure 3b. Finally, the frequency responses were measured and recorded over a frequency range of 2–8 GHz. In order to eliminate the effects of temperature and humidity on the experimental results during the test, all the samples were measured at temperatures and relative humidity ranging from 22.5 °C to 23.0 °C and 45.5–45.8%, respectively. After each RF measurement of the sample, the chip was first flushed several times with phosphate buffer saline (PBS, pH = 6.9, consisting of 137 mmol/L NaCl, 2.7 mmol/L KCl, 10 mmol/L Na_2_HPO_4_, and 2 mmol/L KH_2_PO_4_) and then flushed with DI water prior to drying by air-blowing to ensure the glucose samples are completely removed such that the chip could be used repeatedly for multiple measurements.

## 3. Results and Discussions

### 3.1. Optimization of Sensitivity

**Figure 4:**
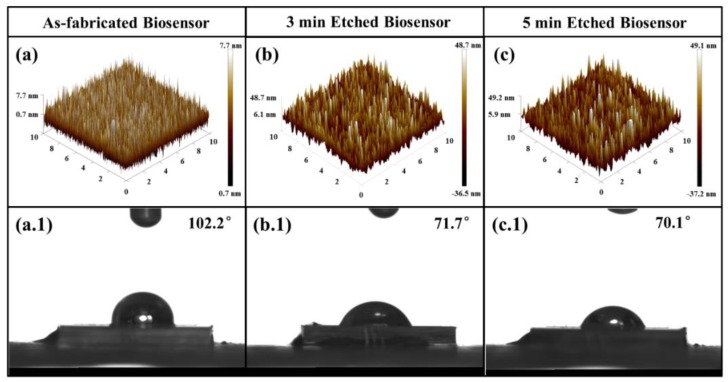
The AFM 3-D view of surface morphologies of top Au layers with (**a**) as-fabricated, (**b**) 3 min etched, and (**c**) 5 min etched. The images of 150 mg/dL glucose droplet onto biosensor with (**a.1**) as-fabricated, (**b.1**) 3 min etched, and (**c.1**) 5 min etched.

To enhance the sensitivity of the biosensor, a series of controlled dry etching processes based on Au layers were systematically carried out on the biosensor to investigate the effect between the roughness of surface and hydrophily hydrophobicity. Figure 4a–c presents the surface roughnesses of the top Au layers measured by atomic force microscope (AFM) which are as-fabricated and etched based on Cl_2_/Ar plasmas processing with 3 min and 5 min, respectively. The parameters of the Au dry etch process are shown in Table 1. As shown in Figure 4a, a smooth surface can be observed on the as-fabricated top layer with a root mean square (RMS) roughness of 2.03 nm. When the chip is treated with Cl_2_/Ar-based plasmas for 3 min, the quality of surface roughness of top layer is deteriorated to 12.3 nm, as depicted in Figure 4b. After dry etching for 5 min, there is no obvious change on the surface roughness showing a RMS of 13.2 nm. We can draw that the extra etching treatment, refering to over 3 min dry etching process, will not induce the Au roughness the deterioration any more after preliminary modification of the surface. Figure 4a.1–c.1 demonstrates the sessile drop data of glucose with 150 mg/dL on biosensor chip surface corresponding to above-mentioned three conditions. It can be observed that as-fabricated chip is highly hydrophobic with a glucose contact angle of ~102.2°. While the contact angle decreases to ~71.7°and ~70.1° after dry etching for 3 min and 5 min, respectively. The results show that the hydrophilicity of the biosensor increases as the surface roughness decreases, suggesting that the surface from hydrophobicity to hydrophilicity can be changed by the deteriorating surface roughness with plasma dry etching in order to promote the rate of fixation of glucose molecules and the resonance process.

### 3.2. Responses of Biosensor

**Figure 5:**
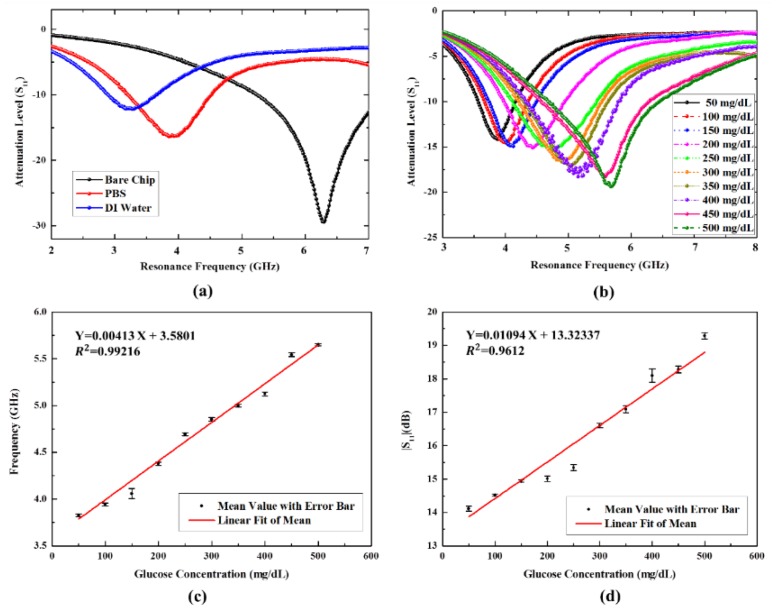
Shift in resonance frequency and magnitude of reflection coefficient (S_11_) under different conditions: (**a**) various solution and (**b**) various glucose samples concentrations (50–500 mg/dL). Regression analysis for the shift in resonance frequency (**c**) and magnitude of reflection coefficient (S_11_) (**d**) with error bars for different glucose sample concentrations.

The responses of the proposed chip can be illustrated using RF parameters. In this work, we first measure the reflection coefficient and the resonance frequency under condition of the bare chip, the chip dropped using PBS solution, and the chip dropped using DI water, respectively. As shown in Figure 5a, the resonance frequency of bare chip is located at 6.30 GHz, and the other resonance frequencies revealed a downward shift with the instillation of PBS solution and DI water, which are located at 3.94 GHz and 3.30 GHz, respectively. Meanwhile, magnitudes of the reflection coefficient show an increasing variation ranging from −29.47 dB to −12.18 dB. The reasons for this can be explained as follows: the viscosity of DI water is lower than that of glucose solution and PBS, but conversely, the effective permittivity of DI water is greater than air, glucose solution, and PBS, resulting in the maximum frequency shift after dripping water onto the chip [51]. Then, a response experiment with different concentrations of glucose ranging from 50 mg/dL up to 500 mg/dL is explored. When the glucose solution is dropped on the surface of bare chip with increasing concentration, the resonance peaks regularly shift upward with an increasing of the magnitude of the reflection coefficient, as a result of the regular variation of effective permittivity, which is depicted in Figure 5b. It can be observed that the chip filled with glucose solution shows a tolerable resonance frequency shift in the range of 3.84–5.62 GHz accompanied with magnitude of reflection coefficient variation from −14.18 dB to −19.40 dB. In comparison to the bare chip, the maximum resonance frequency shift occurs at a glucose level of 50 mg/dL with a shift of 2.46 GHz, and the maximum reflection coefficient variation comes up to 15.29 dB based on the same glucose level.

The relationships between glucose concentration and resonance frequency as well as glucose concentration and magnitude of reflection coefficient are depicted in Figure 5c,d, respectively. The regression analysis can be calculated as follows:

Y_1_ [GHz] = 0.00413 × X [mg/dL] + 3.5801 (R^2^ = 0.99216) (11)

Y_2_ [dB] = 0.01094 × X [mg/dL] + 13.32337 (R^2^ = 0.9612) (12)

where Y_1_, Y_2_, and X represent the resonance frequency, magnitude of reflection coefficient, and glucose concentration, respectively. Interestingly, a good linear correlation between glucose concentration and resonance frequency as well as glucose concentration and magnitude of reflection coefficient can be fitted, which unveils that both resonance frequency shift approach and magnitude of reflection coefficient variation approach can be applied to identify glucose level detection, paving a widening avenue for multi-parameter-sensitive detection of glucose.

### 3.3. Reusability, Real-Time Response Capability and LOD

**Figure 6:**
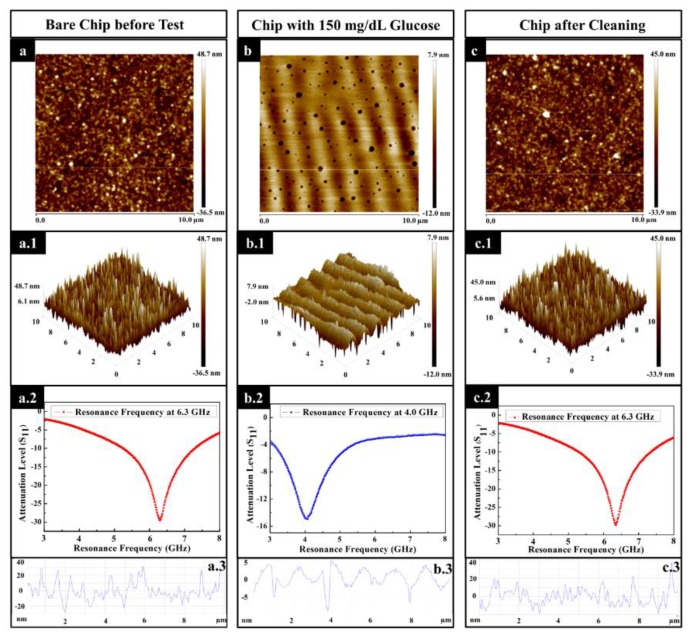
The analysis for surface morphology of the sensing area. (**a**) The 2D view of bare biosensor, (**a.1**) the 3D view of bare biosensor, (**a.2**) the RF measurement of bare biosensor, and (**a.3**) the cross-sectional surface line profile of surface morphology for bare biosensor. Bare chip refers to the chip treated by a 3 min dry etching process. (**b**) The 2D view of biosensor with glucose concentration of 150 mg/dL, (**b.1**) the 3D view of biosensor with glucose concentration of 150 mg/dL, (**b.2**) the RF measurement of biosensor with glucose concentration of 150 mg/dL, and (**b.3**) the cross-sectional surface line profile of surface morphology for biosensor with glucose concentration of 150 mg/dL. (**c**) The 2D view of biosensor after cleaning, (**c.1**) the 3D view of biosensor after cleaning, (**c.2**) the RF measurement of biosensor after cleaning, and (**c.3**) the cross-sectional surface line profile of surface morphology for biosensor after cleaning.

For the purpose of investigating the reusability of the proposed biosensor, the surface morphology of the sensing area of the chip, involving bare chip before test, chip added with 150 mg/dL glucose, and chip after cleaning, were characterized. Figure 6a,a.1,a.2, present the 2D and 3D view of the bare chip with an RMS value of 12.3 nm and a resonance frequency of 6.30 GHz, respectively. When the glucose solution was added to the chip with a concentration of 150 mg/dL, an RMS value of 2.52 nm and a shifted resonance frequency of 4.07 GHz can be achieved, which are shown in Figure 6b,b.1,b.2, respectively. The acquisition of such roughness compared with the bare chip is ascribed to the glucose particles coverage on uneven peaks and valleys of the surface. Interestingly, some cavities and wrinkles appear on the surface of the biosensor as a result of the uneven evaporation rate of the glucose solution. Then, the biosensor is rinsed with PBS and DI water. The RMS value of the biosensor after-cleaning was 11.7 nm (Figure 6c,c.1) and the resonance frequency returned to 6.30 GHz (Figure 6c.2). From another perspective, the surface roughness profiles, before the test (Figure 6a.3) and after cleaning (Figure 6c.3), were found to be similar, which is obviously different from the conditions under the test (Figure 6b.3). The above-discussed results provide a good illustration that there is no chemical immobilization involved for the chip to create a resonance frequency and return the initial value after the test. Therefore, the proposed biosensor has a property of reusability for the future application.

The real-time response capability of the biosensor is very valuable for clinical medical applications. Therefore, based on the experimental setup shown in Figure 3a, we characterized the real-time response capability of a 3 min-etched chip by measuring the time at which the output waveform changes since glucose solution with concentration of 150 mg/dL is dropped onto the chip. It can be observed that the output waveform changes within 0.85 s. The obtained quick response time demonstrates that the proposed biosensor has an excellent real-time response capability. Because of the limitation of the VNA scanning speed, which is 0.85 s/point, the real-time response of the biosensor is superior to 0.85 s. With regard to the LOD, it is calculated from the following equation [52]:

LOD = 3.3 × SD/m(13)

where SD stands for the standard deviation of the frequency response and m denotes the slope of the regression line. To obtain the standard deviation of the frequency response and slope of the regression line, the variations of resonance frequency during the five tests were measured, and the results are summarized in Table 2. With the aid of measurement, the LOD of 8.01 mg/dL was calculated. All relative standard deviations of less than 1% indicate a small discrete with respect to each resonance frequency. A comparative analysis based on RF techonology is summarized in Table 3, where the response time and LOD are superior to those achieved in previous studies.

## 4. Conclusions

Benefiting from an optimized integrated passive device fabrication technique, a novel non-invasive RF glucose sensor was successfully designed, fabricated, and studied with the merits of quantitative detection, robust reusability, ultrafast response time (<0.85 s) and ultralow LOD (8.01 mg/dL). Based on the EM simulation result, it is verified that the embedded hammer-shaped capacitors had a fatal effect on the resonance frequency. Meanwhile, the phenomenon that the concentrations of glucose are proportional to biosensor resonance frequency and the magnitude of reflection coefficient enables the proposed chip to accurately detect the glucose levels in diabetes patients. In order to enhance the sensitivity of the biosensor, a Cl_2_/Ar plasma-based dry etching treatment with different etching times was carried out, which implies that etching treatment with appropriate time can modify the metal surface to a certain extent. The above findings do not merely reveal several new approaches to realize a stable and accurate platform for the detection of glucose levels but also provide a promising approach for the detection of glucose levels in human serum.

## Figures and Tables

**Table 1:** The recipe of Au dry etch process parameters.

Recipe Set Values	
Cl_2_	20 sccm
Ar	5 sccm
Pressure	2 mTorr
RF coil power	600 W
RF platen power	250 W
Temperature	20 °C

**Table 2:** Performances of the different measured resonance frequencies with various glucose concentrations ranging from 50 mg/dL to 500 mg/dL.

S.N ^1^	G. C. ^2^ (mg/dL)			Resonance Frequency (GHz)					RSD ^5^ (%)
		1st Test	2nd Test	3rd Test	4th Test	5th Test	Mean ^3^	Mean ± RSD (f_av_^4^)	
1	50	3.83	3.84	3.81	3.83	3.82	3.826	3.826 ± 0.26%	0.26
2	100	3.94	3.98	3.93	3.93	3.93	3.942	3.942 ± 0.49%	0.49
3	150	4.08	4.03	4.06	4.05	4.07	4.058	4.058 ± 0.42%	0.42
4	200	4.38	4.40	4.36	4.38	4.37	4.378	4.378 ± 0.30%	0.30
5	250	4.72	4.68	4.67	4.70	4.70	4.694	4.694 ± 0.37%	0.37
6	300	4.85	4.84	4.87	4.83	4.87	4.852	4.852 ± 0.32%	0.32
7	350	5.00	4.97	4.99	5.01	5.02	4.998	4.998 ± 0.34%	0.34
8	400	5.10	5.17	5.19	5.06	5.10	5.124	5.124 ± 0.94%	0.94
9	450	5.54	5.53	5.56	5.53	5.56	5.544	5.544 ± 0.24%	0.24
10	500	5.63	5.64	5.66	5.66	5.66	5.650	5.650 ± 0.22%	0.22

^1^ sample number; ^2^ glucose concentration; ^3^ average of the five experiments; ^4^ final average resonance frequency; ^5^ relative standard deviation.

**Table 3:** Performance of the proposed glucose sensor compared with recently reported studies.

Ref.	Proposed Method	Response Time (s)	LOD (mg/dL)
[52]	RF patch on silicon substrate by characterization of effective dielectric constant	Not Given	26.54
[53]	Magnetic acoustic resonance sensor	22 min	36.36
[54]	Impedance spectroscopy	Not Given	12.02
[55]	Forster resonance energy transfer	15 min	25
[56]	Rectangular meandered line based RF resonator	60	8.01
[57]	Air bridge enhanced capacitor based RF resonator	40	9.69
This work	hammer-shaped capacitors and spiral inductors constructed RF resonator	<0.85 s	8.01
